# Solitary Giant Primary Gastric Plasmacytoma Mimicking Gastric Adenocarcinoma In Situ

**DOI:** 10.7759/cureus.68674

**Published:** 2024-09-04

**Authors:** Sergio A Bolivar, Patricia Medina, Maria Cynthia Fuentes, Humberto Martinez-Cordero, German Salguedo

**Affiliations:** 1 Internal Medicine, Hospital Militar Central, Bogotá, COL; 2 Hematology, Hospital Militar Central, Bogotá, COL; 3 Hematology and Oncology, Hospital Militar Central, Bogotá, COL

**Keywords:** hemato-oncology, plasma cell neoplasms, adenocarcinoma, immunohistochemistry, primary gastric plasmacytoma

## Abstract

A solitary extraosseous plasmacytoma is a rare type of plasma cell neoplasm. Its occurrence in the stomach is particularly unusual and can easily be mistaken for more common types of tumors. We describe a case involving a solitary extraosseous plasmacytoma in a patient who experienced weight loss as the sole symptom. Initially, the condition was misdiagnosed as gastric adenocarcinoma based on endoscopic biopsy results, leading to a gastrectomy after neoadjuvant chemotherapy. Subsequent examination of the pathological specimen revealed the presence of plasma cell neoplasia alongside a gastric adenocarcinoma in situ.

## Introduction

Solitary plasmacytoma (SP) is an infrequent condition marked by the localized growth of monoclonal plasma cells. There are two identified forms of localized plasma cell neoplasms: solitary plasmacytoma of bone (SBP) and primary extraosseous/extramedullary plasmacytoma (EMP). EMP accounts for only 3-5% of plasma cell malignancies and typically occurs at extranodal sites, with 90% of cases found in the head and neck region [1]. Gastrointestinal involvement is rare, representing less than 5% of all EMP cases [2]. This entity is usually localized but may progress to multiple myeloma depending on marrow involvement, with a 10% risk of progression to multiple myeloma at three years if no clonal plasma cells are present in the bone marrow, and a 20% risk of progression at three years if there is minimal marrow involvement (<10% clonal plasma cells)[2]. The most common clinical presentation is gastrointestinal bleeding or peptic ulcers that do not improve with conventional treatment [3,4,5].

Herein, we report a case of a patient initially diagnosed with gastric adenocarcinoma. Surgical pathology unexpectedly revealed dual primary tumors: gastric adenocarcinoma and gastric plasmacytoma (GP). The latter is an example of primary extraosseous plasmacytoma (EMP), illustrating the importance of considering EMP in differential diagnoses of gastric tumors.

## Case presentation

**Figure 1:**
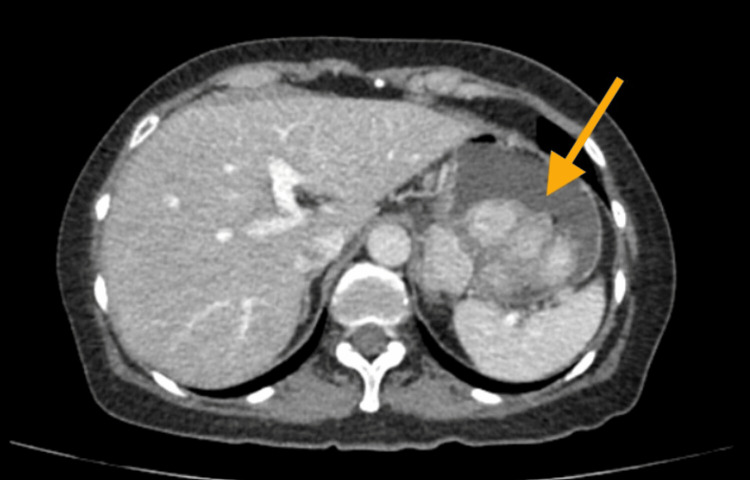
Contrast-enhanced abdominal computed tomography (CT) A mass can be observed in the posteromedial wall of the gastric fundus, multilobulated and enhanced with contrast medium, reaching dimensions of 42 × 52 × 60 mm, indicated by the orange arrow.

A 63-year-old female presented with recent-onset epigastric pain accompanied by unintentional weight loss of 2 kg. She denied any associated gastrointestinal symptoms, including changes in bowel habits, nausea, vomiting, or signs of gastrointestinal bleeding. Her vital signs were within normal limits. The abdominal examination was normal, with no masses or adenopathy palpated. Her past medical history was unremarkable, with no known chronic illnesses or prior significant medical conditions. A contrast-enhanced abdominal CT scan was performed, revealing a solid mass suggestive of malignancy in the posteromedial wall of the gastric fundus, accompanied by nearby neoplastic lymphadenopathy. Figure 1 illustrates a contrast-enhanced abdominal computed tomography (CT) scan.

An upper gastrointestinal endoscopy was conducted, revealing subepithelial solid masses in the gastric fundus. Biopsies were taken during the procedure, and subsequent histopathological examination confirmed the presence of tubular gastric adenocarcinoma stage IIA disease. *Helicobacter pylori* testing yielded negative results. The patient underwent a perioperative FLOT chemotherapy (docetaxel, oxaliplatin, and fluorouracil/leucovorin) followed by gastrectomy.

**Figure 2:**
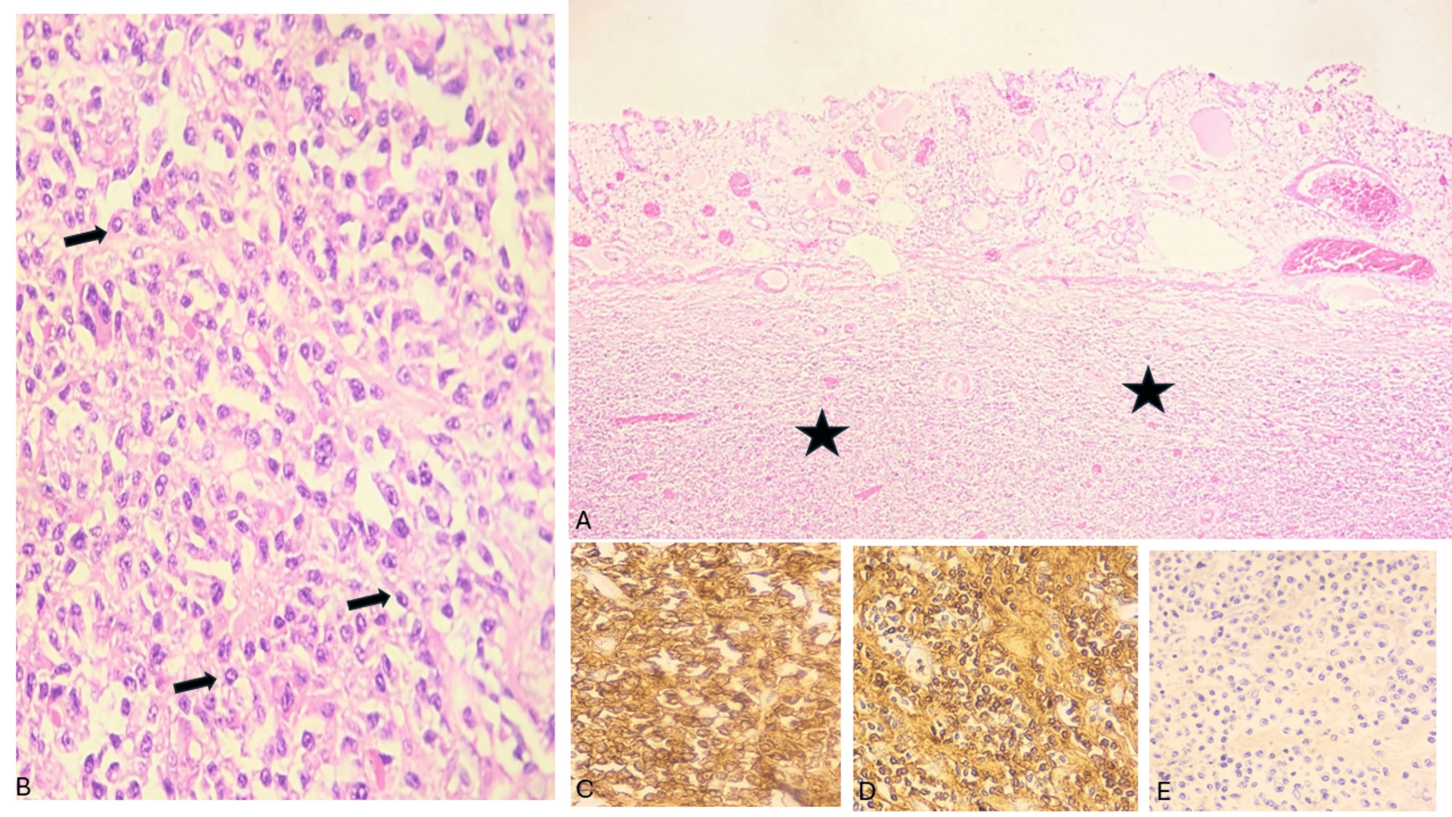
Morphological and immunohistochemical analysis of the tumor lesion Histologic sections show a gastric wall with a diffuse infiltrate of neoplastic cells below the muscularis mucosae (stars) (A). Neoplastic cells have plasmacytic morphology with eccentric nuclei and abundant amphophilic cytoplasm (black arrows) (B). Immunohistochemistry reveals strong expression of CD38 confirming plasmacytic lineage (C), with monotypic expression of kappa light chain (D), without expression of lambda light chain (E).

In the surgical pathology examination, surprisingly, high-grade gastric dysplasia/adenocarcinoma in situ (tumor size: 0.5 cm in largest diameter) without infiltrating component was found, coexisting with morphological and immunohistochemical findings compatible with involvement by plasma cell neoplasia (tumor size approximately: 11 x 7 x 4 cm) and six out of 21 perigastric lymph nodes involved by plasma cell neoplasia. Immunohistochemical analysis of the tumor cells showed positivity for CD38 and monotypic expression of a kappa light chain, without an expression of a lambda light chain; confirming plasmacytic lineage. As shown in Figure 2, a detailed morphological and immunohistochemical analysis of the tumor lesion was performed.

Serum protein electrophoresis showed a monoclonal protein peak. Molecular analysis revealed deletion of TP53 and rearrangement of IgH, indicative of high-risk neoplasia associated with adverse prognosis and increased mortality.

Furthermore, serum and urine immunofixation assays detected monoclonal protein band IgG kappa. Bone marrow biopsy yielded negative results for clonal plasma cells or other aberrations, no lytic lesions were observed on the low-dose whole-body CT scan, and there were no findings of hypercalcemia or anemia.

Following gastrectomy, an 18-FDG PET/CT scan was performed, revealing no evidence of infiltrative pathology at the surgical site or elsewhere anatomically; also, an immunofixation assay was negative for monoclonal protein bands. Considering the primary nature of the completely resected GP and the absence of clonal plasma cell involvement in the bone marrow, the patient was deemed to be in remission. However, stringent monitoring with serum protein electrophoresis with immunofixation, and 18-FDG PET/CT scan was also recommended for ongoing surveillance.

## Discussion

The importance of defining active treatment versus monitoring lies in the fact that patients with SP have a higher risk of developing symptomatic multiple myeloma (MM) in the next 10 years (60% SBP and 20% EMP) [4,6]. One of the prognostic factors in SBP for progression to MM is bone marrow infiltration by clonal plasma cells at diagnosis. Another predictor of progression is the presence at diagnosis of serum-free light chains (sFLC) and positivity after treatment. Progression of an SP is defined as local relapse, the appearance of additional lesions without signs of MM, or progression to MM [4]. Table 1 presents the diagnostic criteria for SB and related disorders.

**Table 1:** Diagnostic criteria for solitary plasmacytoma and overlapping disorders EMP: extraosseous/extramedullary plasmacytoma, PC: plasma cell, BM: bone marrow, MM: multiple myeloma, SBP: solitary bone plasmacytoma, PET-CT: positron emission tomography-computed tomography Adapted from [7]

		Plasmacytoma	Serum monoclonal protein	Bone marrow	End organ damage	Work-up PET-CT	Risk of progression to MM	Primary Treatment	Additional Treatment Options
Solitary plasmacytoma	Solitary plasmacytoma	Present	Not required for diagnosis, but a monoclonal protein may be present.	Negative	Not required	No other lesions	10% will progress to MM within 3 years	Local radiotherapy	Surgery (if accessible and resectable)
	Solitary plasmacytoma with minimal bone marrow involvement	Present	Not required for diagnosis, but a monoclonal protein may be present.	Monoclonal PC infiltration < 10%	Absent	No other lesions	60% with SBP or 20% with EMP will progress to MM within 3 years	Local radiotherapy and close monitoring	Chemotherapy (if progression occurs)
Multiple myeloma	Macrofocal myeloma	Present and multiple	Not required for diagnosis, but a monoclonal protein may be present.	Monoclonal PC infiltration < 10%	Possible but Limited systemic symptoms	Multiple bone or other organ lesions	30% to 50% over a period of 2 to 5 years.	Combination chemotherapy	Autologous stem cell transplant, radiotherapy for focal lesions
	Multiple myeloma	Not required but can occur	Present but may be absent in the case of non-secretory myeloma	Monoclonal PC infiltration ≥ 10%	Present	Multiple bone or other organ lesions	Meets criteria for multiple myeloma	Combination chemotherapy	Autologous stem cell transplant

Most patients with GPs are elderly with nonspecific symptoms, including anorexia, weight loss, epigastric discomfort, or gastrointestinal bleeding [6]. During endoscopy, GPs often appear as nodular masses, which typically leads to differential diagnoses such as gastrointestinal stromal tumors (GISTs) or lymphomas. Frequently, the findings on endoscopy simulate a carcinoma when the mass is ulcerated or a linitis plastica when mucosal folds are diffusely thickened [6,7].

Among the therapeutic possibilities for a solitary SP is fractionated localized radiotherapy with doses between 40-50 Gy, this being currently the standard of care, with good response rates evidenced in retrospective analyzes, but without randomized controlled trials comparing this approach with best supportive care or chemotherapy. Another therapeutic option is surgical resection, usually followed by radiotherapy due to the risk of relapse. This option is recommended in SBP only if a surgical approach is required due to bone pathology. In EMP, it is recommended only in cases of defined, large lesions. Chemotherapy as a post-radiation adjuvant treatment is controversial [7,8].

Post-treatment follow-up is recommended in the case of measurable diseases according to the International Myeloma Working Group (IMWG) criteria; in the case of non-measurable diseases, the Response Evaluation Criteria in Solid Tumours (RECIST) criteria are added [7]. Table 2 presents case reports of plasmacytomas located in the gastrointestinal tract, detailing their characteristics and treatment approaches. We have compared the management of our patient with these standard approaches documented in the literature, and as shown in Table 2, our approach is aligned with established practices. This is the first reported case of the coexistence of gastric adenocarcinoma in situ and GP. To date, no standard clinical care protocol for this combination is defined in the literature. For gastric adenocarcinoma in situ, treatment typically involves endoscopic resection or surgical intervention, depending on the extent and localization of the disease. However, the management of cases with concurrent plasmacytoma is not well established.

**Table 2:** Published case reports of intra-abdominal extramedullary plasmacytoma

Study	Country	Patient		Anatomical location	Presentation	Therapy	
		Age (years)	Sex			Operative	Non-operative
Park et al., 2014 [9]	South Korea	70	Male	Stomach	Dyspepsia	Endoscopic submucosal resection	Thalidomide, dexamethasone
Zhao et al., 2014 [10]	China	79	Male	Stomach	Epigastric pain	Total gastrectomy, subtotal pancreatectomy, splenectomy	None
Han et al., 2014 [11]	South Korea	62	Male	Stomach	Periumbilical pain	Gastric bypass (palliative)	None
Fukuhara et al., 2016 [12]	Japan	36	Male	Stomach	Dyspnea, fatigue	Total gastrectomy, extended lymphadenectomy	Bortezomib, cyclophosphamide, dexamethasone
Ammar et al., 2010 [13]	USA	69	Female	Duodenum	Fatigue, melaena	Percutaneous transhepatic biliary drainage	Extracorporeal radiotherapy
Ariyarathenam et al., 2013 [14]	UK	58	Male	Ileum	Intussusception, small bowel obstruction	Segmental small bowel resection	None
Gabriel et al., 2014 [15]	USA	62	Male	Ileum, caecum	Melaena	Endoscopic submucosal resection (failed), right hemicolectomy	None
Weidenbaum, 2020 [16]	USA	83	Female	Stomach	Anemia	None	Extracorporeal radiotherapy and bortezomib, cyclophosphamide, dexamethasone
Jeong Lee et al., 2020 [17]	South Korea	63	Female	Stomach	Abdominal pain	Surgical resection	None
Vara-luis et al., 2024 [18]	Portugal	71	Female	Stomach	Dyspepsia	Surgical resection	None

## Conclusions

The simultaneous presentation of gastric adenocarcinoma in situ and primary gastric extraosseous plasmacytoma is unprecedented in the medical literature, underscoring the exceptional nature and clinical importance of this case. There is no consensus on a standardized treatment protocol for EMP. Existing studies indicate that the most common treatments are surgery or radiation therapy, sometimes combined with chemotherapy. In addition, there are no long-term follow-up studies specifically focusing on GPs.
